# Intrabacterial Regulation of a Cytotoxic Effector by Its Cognate Metaeffector Promotes Legionella pneumophila Virulence

**DOI:** 10.1128/msphere.00552-22

**Published:** 2023-01-04

**Authors:** Deepika Chauhan, Ashley M. Joseph, Stephanie R. Shames

**Affiliations:** a Division of Biology, Kansas State University, Manhattan, Kansas, USA; b Department of Microbiology and Molecular Genetics, Michigan State University, East Lansing, Michigan, USA; University of Kentucky

**Keywords:** *Legionella pneumophila*, metaeffector, effector functions, virulence

## Abstract

Legionella pneumophila is a natural pathogen of unicellular protozoa that can opportunistically infect macrophages and cause Legionnaires’ Disease. Intracellular replication is driven by hundreds of bacterial effector proteins that are translocated into infected host cells by a Dot/Icm type IV secretion system. L. pneumophila effectors are temporally regulated in part by a unique family of translocated regulatory effectors, termed metaeffectors, which bind and modulate the function of a cognate effector in host cells. Regulation of the cytotoxic effector SidI by its cognate metaeffector, MesI, is critical for L. pneumophila virulence in natural and opportunistic hosts. MesI binds and negatively regulates SidI activity *in vitro*, but how impaired regulation of SidI impairs L. pneumophila intracellular replication is unclear. Using a chromosomally encoded inducible expression system, we found that SidI was toxic to L. pneumophila when uncoupled from MesI. SidI enzymatic activity was required for intrabacterial toxicity since L. pneumophila growth was unaffected by induced expression of a catalytically inactive *sidI* allele. We also found that MesI translocation into host cells was dispensable for intracellular replication and that MesI-deficient bacteria were rapidly degraded within host cells. These data suggest that MesI promotes L. pneumophila intracellular replication by regulating SidI within the bacterium and reveal a unique role for intrabacterial effector regulation by a translocated metaeffector in L. pneumophila virulence.

**IMPORTANCE** Legionella pneumophila replicates within phagocytic host cells using hundreds of effector protein virulence factors, which canonically subvert the function of host proteins and pathways. L. pneumophila encodes a unique family of translocated effectors called metaeffectors, which bind and regulate the function of a cognate effector in host cells. The metaeffector MesI promotes L. pneumophila virulence by regulating the cytotoxic effector SidI; however, the MesI regulatory mechanism is poorly understood. We discovered a unique intrabacterial role for MesI in L. pneumophila virulence. When uncoupled from MesI, SidI was toxic to L. pneumophila *in vitro* and triggered robust bacterial degradation in host cells. Furthermore, translocation of MesI was dispensable for intracellular replication, demonstrating that intrabacterial regulation of SidI contributes to L. pneumophila virulence. These data show a novel and important role for translocated effector activity within the bacterium, which challenges the dogma that L. pneumophila effectors function exclusively within host cells.

## INTRODUCTION

Legionella pneumophila is ubiquitous in freshwater environments where it parasitizes and replicates within free-living protozoa. Coevolution with environmental phagotrophs have conferred on L. pneumophila the ability to replicate within mammalian macrophages, which results in a severe inflammatory pneumonia called Legionnaires’ Disease (1–3). Within host cells, L. pneumophila replicates within an endoplasmic reticulum (ER)-derived compartment called the *Legionella*-containing vacuole (LCV), which avoids endocytic maturation and fusion with lysosomes (4, 5). Biogenesis of the LCV is facilitated through the activity of over 300 effector proteins, bacterial virulence factors translocated into host cells through a Dot/Icm type IV secretion system (T4SS) (6). Dot/Icm-translocated effectors are critical for L. pneumophila virulence in natural and opportunistic hosts (7); however, the role of most effectors remains poorly understood due primarily to functional redundancy and rarity of virulence phenotypes in laboratory infection models.

Many Gram-negative bacterial pathogens employ effector proteins as part of their virulence strategy. Canonically, effector proteins function by targeting host proteins and subverting cellular pathways to the benefit of the pathogen; however, L. pneumophila encodes a unique family of translocated effectors termed metaeffectors, which bind and regulate other L. pneumophila effectors within host cells (8, 9). Metaeffectors are a key component of L. pneumophila pathogenicity since several are required for intracellular replication, but how most metaeffectors promote virulence is poorly understood.

We discovered that regulation of the effector SidI (Lpg2504) by its cognate metaeffector, MesI (Lpg2505), is critical for L. pneumophila virulence (10). Like most L. pneumophila effectors, loss of SidI has no effect on virulence in laboratory infection models; however, loss-of-function mutation in *mesI* (Δ*mesI*) uniquely attenuates L. pneumophila virulence in amoebae, macrophages, and mouse models of Legionnaires’ Disease only when SidI is produced (10). SidI is a cytotoxic GDP-mannose-dependent glycosyl hydrolase that potently blocks eukaryotic mRNA translation (11, 12). MesI binds SidI with high affinity, suppresses its toxicity, and abrogates SidI-mediated translation inhibition *in vitro* (12). The requirement for MesI is relieved when *sidI* is deleted (Δ*sidI*); however, a single amino acid substitution at Arg453, which renders SidI nontoxic and catalytically inactive (11, 12), is also sufficient to restore replication of MesI-deficient L. pneumophila (10). These data have provided key biochemical insights into the MesI regulatory mechanism, but how MesI regulation of SidI promotes intracellular replication is still unclear.

In this study, we made the surprising discovery that MesI promotes L. pneumophila virulence by regulating SidI intrabacterially. SidI was toxic to L. pneumophila when uncoupled from MesI *in vitro*. We found that MesI translocation was dispensable for L. pneumophila intracellular replication and MesI-deficient bacteria are rapidly degraded in host cells. These data suggest a unique and important role for interkingdom effector regulation in L. pneumophila virulence.

## RESULTS

### Overexpression of *sidI* impairs L. pneumophila growth *in vitro*.

**FIG 1:**
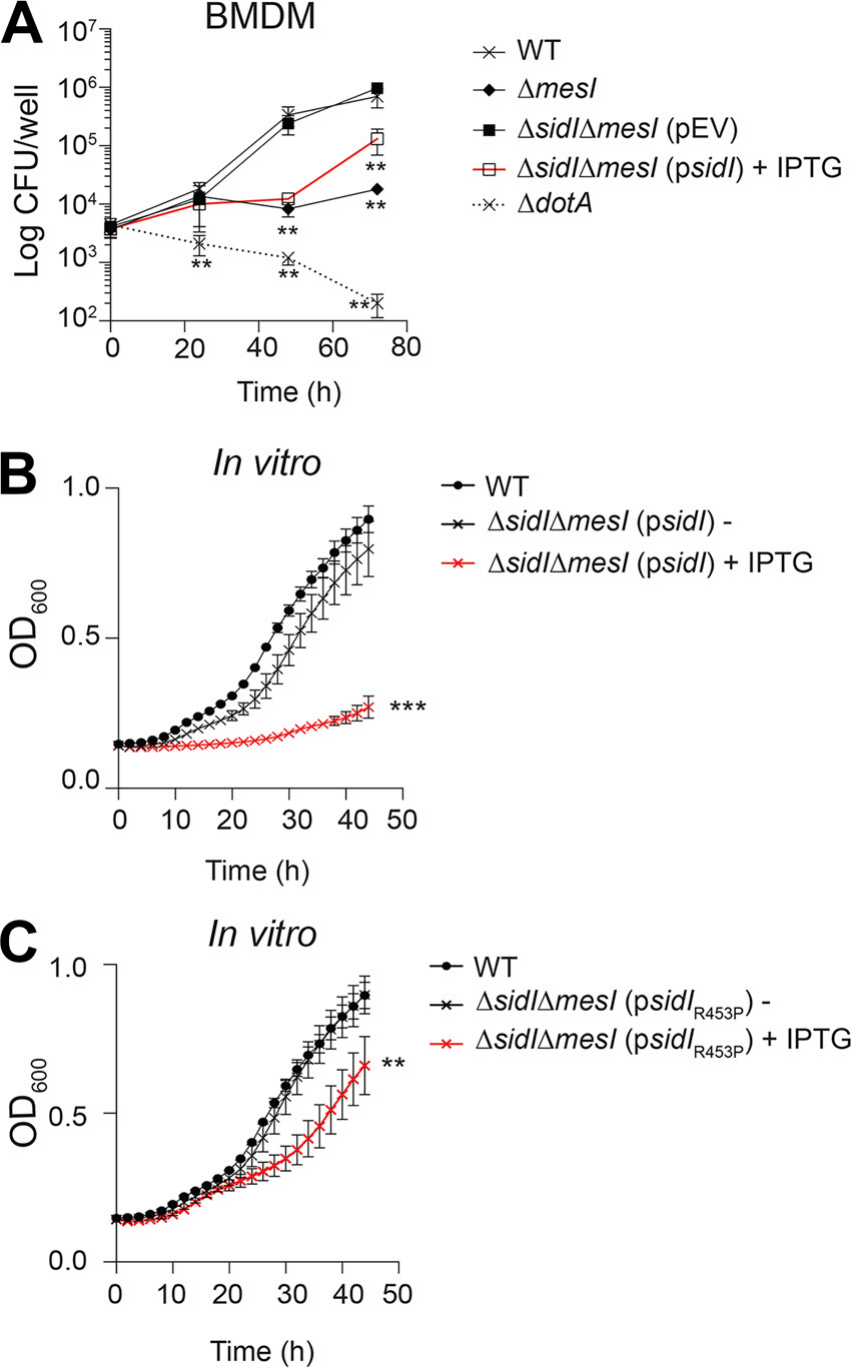
Overexpression of *sidI* attenuates L. pneumophila replication. (A) Fold replication of L. pneumophila strains within *Nlrc4*^−/−^ BMDMs over 72 h. Where indicated, infections were performed in the presence of 1 mM IPTG. Data shown are mean ± SD on samples in triplicate, and asterisks denote statistical significance by Student's *t* test (**, *P *< 0.01). (B, C) Optical density at 600 nm (OD_600_) of L. pneumophila strains grown in AYE broth. Where indicated, bacteria were grown in the presence (+) or absence (−) of 1 mM IPTG. WT growth from the same experiment shown in both panels B and C for comparison. Data shown are mean ± SD on samples in triplicate, and asterisks denote statistical significance by one-way analysis of variance (ANOVA) (**, *P *< 0.01; ***, *P *< 0.0001). Data shown are representative of at least three independent experiments.

We discovered that the L. pneumophila metaeffector MesI promotes bacterial intracellular replication by regulating its cognate effector SidI (10), but how loss of SidI regulation attenuates L. pneumophila virulence is unclear. The L. pneumophila Δ*mesI* strain is severely impaired for intracellular replication, but virulence is restored by *sidI* chromosomal deletion (Δ*sidI* Δ*mesI*) or by a single amino acid substitution (R453P) that renders SidI nontoxic and severely attenuates catalytic activity (*sidI*_R453P_Δ*mesI*) (10–12). We initially validated that excess SidI activity impairs L. pneumophila intracellular replication by plasmid-based genetic complementation of the Δ*sidI* mutation. We infected primary bone marrow-derived macrophages (BMDMs) from *Nlrc4*^−/−^ mice, which are permissive to flagellated L. pneumophila (13, 14), and quantified CFU over 72 h of infection. Indeed, induced expression of *sidI* from a complementing plasmid (p*sidI*) severely attenuated L. pneumophila replication within BMDMs, similar to what we observed for the L. pneumophila Δ*mesI* strain (Fig. 1A). Interestingly, we had to induce *sidI* expression at the time of infection since we were unable to culture the L. pneumophila Δ*sidI* Δ*mesI* (p*sidI*) strain on solid inducing media. Inducible plasmid-based genetic complementation is an established technique to fulfill molecular Koch’s postulates, and we routinely culture L. pneumophila on inducing media (10, 12, 15–17). We hypothesized that *sidI* adversely affects L. pneumophila fitness and found that replication of the L. pneumophila Δ*sidI* Δ*mesI* strain harboring p*sidI* was unable to replicate in broth under inducing conditions (+isopropyl-β-d-thiogalactopyranoside [IPTG]) compared to both wild-type (WT) L. pneumophila and the Δ*sidI* Δ*mesI* (p*sidI*) strain grown under noninducing conditions (Fig. 1B). Bacteria harboring p*sidI* grown under noninducing conditions were slightly attenuated for replication, but the differences did not reach statistical significance (Fig. 1B). Induced expression of the *sidI*_R453P_ allele also significantly impaired L. pneumophila growth (Fig. 1C); however, growth attenuation was far less severe than that observed for bacteria expressing the wild-type *sidI* allele (Fig. 1B). SidI_R453P_ retains a small amount activity (11, 12), which is likely responsible for the attenuation observed in Fig. 1C. These data support previous observations that SidI activity is dose dependent. These data also correlate with our previous observation that the L. pneumophila Δ*mesI* strain is not attenuated for growth in broth (10). Since endogenous *sidI* expression is >3-fold lower in broth compared to bacteria grown within host cells (18), the amount of endogenous SidI produced in broth is likely insufficient to impair L. pneumophila growth. Plasmid-based overexpression of an effector gene has not previously been associated with impaired bacterial growth *in vitro*, and these data suggest that SidI confers a dose-dependent fitness disadvantage on L. pneumophila when stoichiometrically uncoupled from MesI.

### MesI rescues the SidI-mediated growth defect *in vitro*.

**FIG 2:**
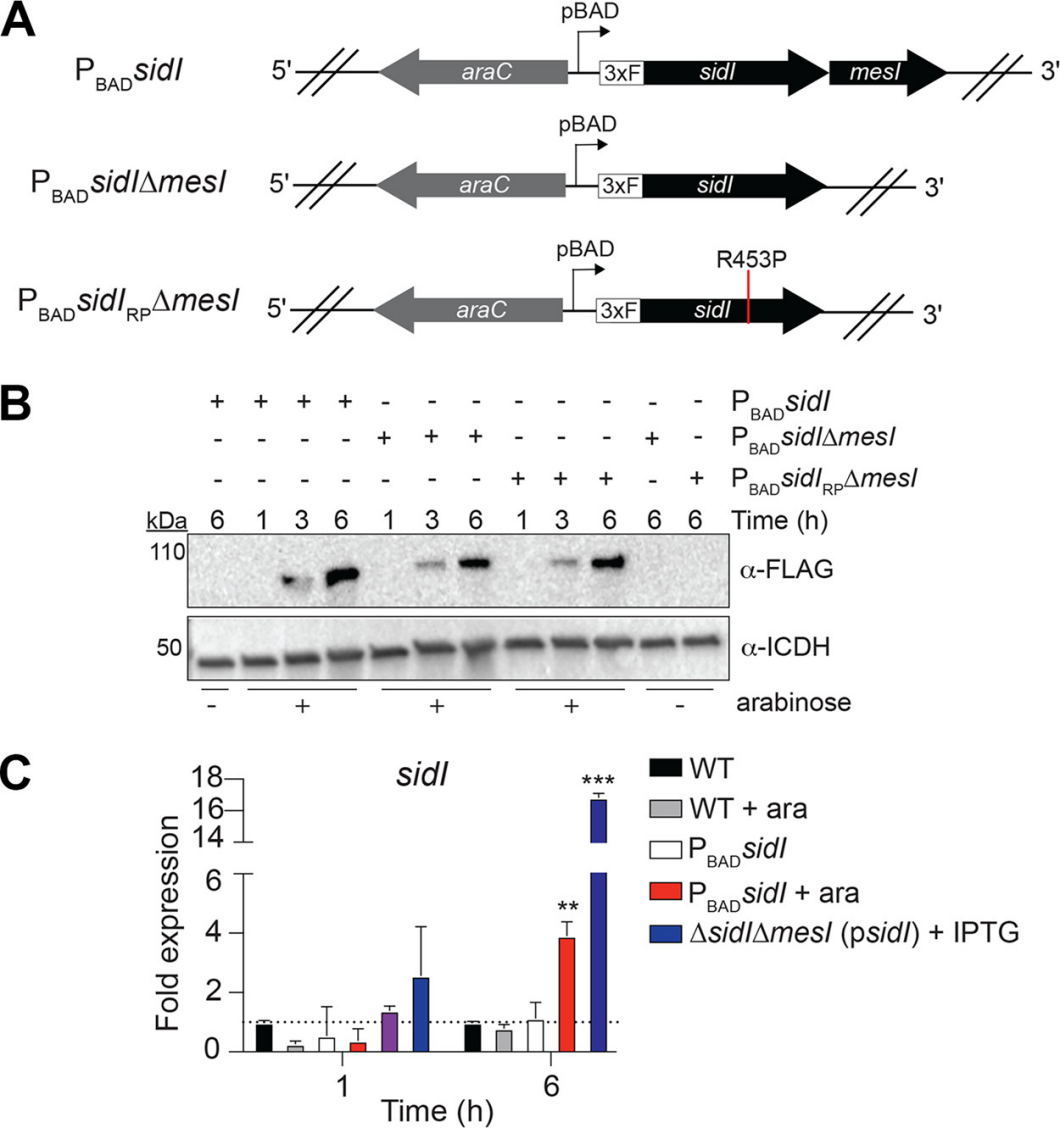
Establishment of a chromosomal arabinose-inducible expression system in L. pneumophila. (A) Schematic showing P_BAD_ insertion upstream of the *sidI* locus in the L. pneumophila chromosome. 3×F, in-frame *3×flag* epitope tag. (B) Western blot showing 3xFLAG-SidI production in the indicated P_BAD_ strains in the presence (+) or absence (−) of arabinose (ara; 0.6% [wt/vol]) after growth in AYE broth for 1, 3, or 6 h. (C) Quantitative RT-PCR for expression of *sidI* grown for 1 or 6 h in AYE broth. Fold expression in OD-normalized cultures (*n *= 3) was calculated relative to wild-type L. pneumophila at each time point. Asterisks denote statistical significance by Student's *t* test (***, *P *< 0.001; **, *P *< 0.01; compared to WT) on samples in triplicate. Data are representative of at least two independent experiments.

MesI suppresses SidI toxicity in yeast (10); thus, we hypothesized that MesI also suppresses SidI toxicity in L. pneumophila. To test this hypothesis, we generated L. pneumophila strains that enable tightly controlled inducible expression of the *sidI-mesI* locus from its endogenous position in the chromosome. We inserted the *araC*-*araBAD* (P_BAD_) promoter and an in-frame *3×flag* epitope tag directly upstream of *sidI* in the chromosome of the L. pneumophila WT, Δ*mesI*, and *sidI*_R453P_Δ*mesI* strains (Fig. 2A). Kinetics and abundance of 3xFLAG-SidI fusion protein production were consistent between strains and only observed under arabinose-inducing conditions (Fig. 2B). We observed a tightly controlled increase in P_BAD_-mediated *sidI* expression relative to endogenous levels (Fig. 2C). The ~4-fold increase in *sidI* expression from the P_BAD_ promoter is more physiologically relevant than the >10-fold increase in expression from the P*tac* promoter (Fig. 2C) (18). Thus, we have established L. pneumophila strains in which we can control expression of *sidI* and *mesI* in their native stoichiometry and endogenous locus in the chromosome.

**FIG 3:**
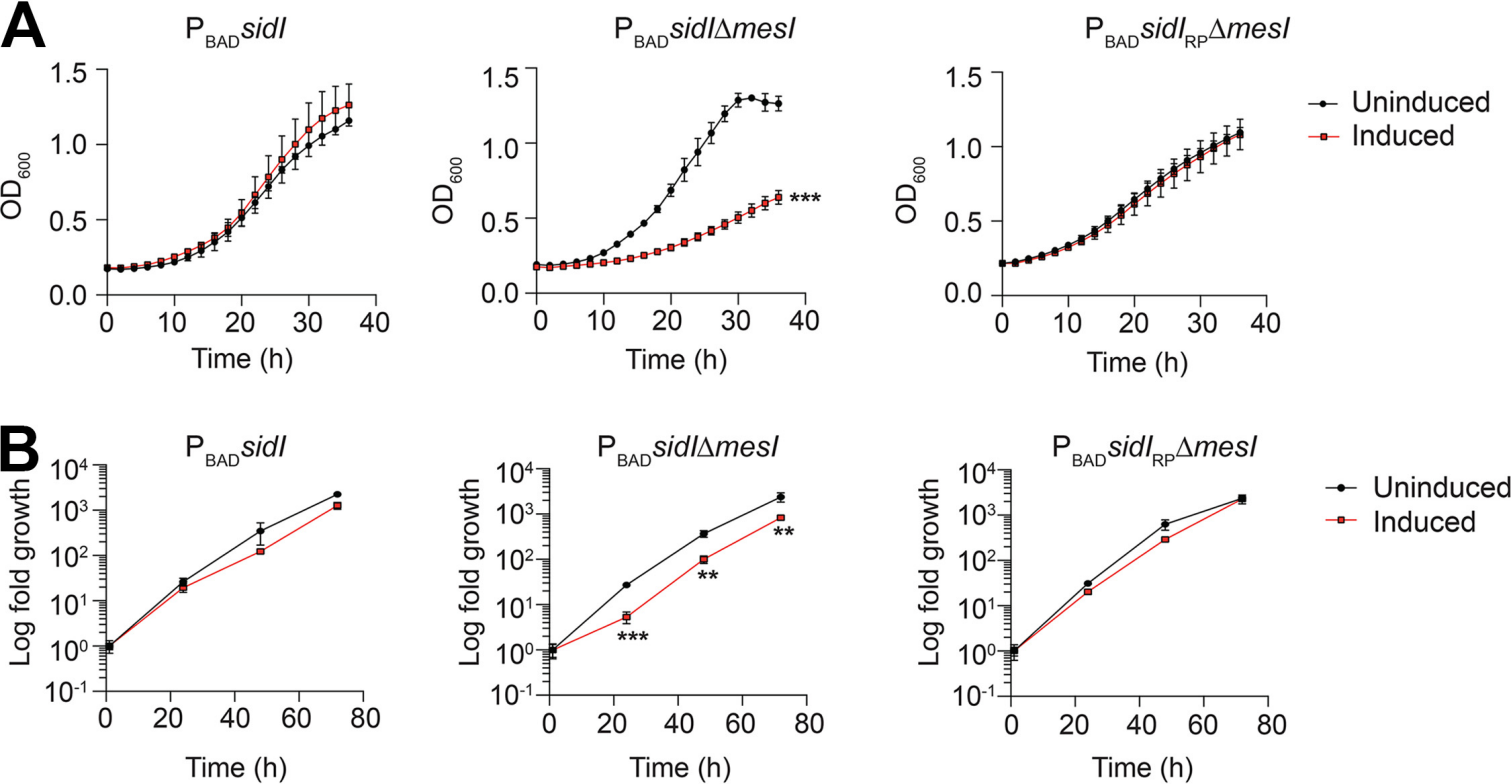
SidI impairs L. pneumophila growth when uncoupled from MesI. (A) Replication of the L. pneumophila P_BAD_ strains grown in AYE broth *in vitro* under inducing (1% arabinose [wt/vol]) or noninducing conditions. Data shown are mean ± SD. OD_600_ values from samples in triplicates and asterisks denote statistical significance by Student's *t* test (**, *P *< 0.01). (B) Fold replication of L. pneumophila strains within *Nlrc4*^−/−^ BMDMs under inducing (2% arabinose [wt/vol]) or noninducing conditions. Data shown are mean ± SD on samples in triplicates, and asterisks denote statistical significance by Student's *t* test (**, *P *< 0.01). Data shown are representative of at least three independent experiments.

We leveraged our L. pneumophila P_BAD_ strains (Fig. 2A) to test the hypothesis that stoichiometric production of MesI abrogates SidI-mediated growth attenuation. We quantified replication our L. pneumophila P_BAD_ strains under inducing and noninducing conditions and found that induced expression of *sidI*, but not the inactive *sidI*_R453P_ allele, significantly attenuated bacterial growth only when uncoupled from *mesI* in broth culture (Fig. 3A) and within BMDMs (Fig. 3B). Thus, the SidI-mediated growth defect is suppressed by MesI.

### MesI suppresses SidI intrabacterial toxicity.

**FIG 4:**
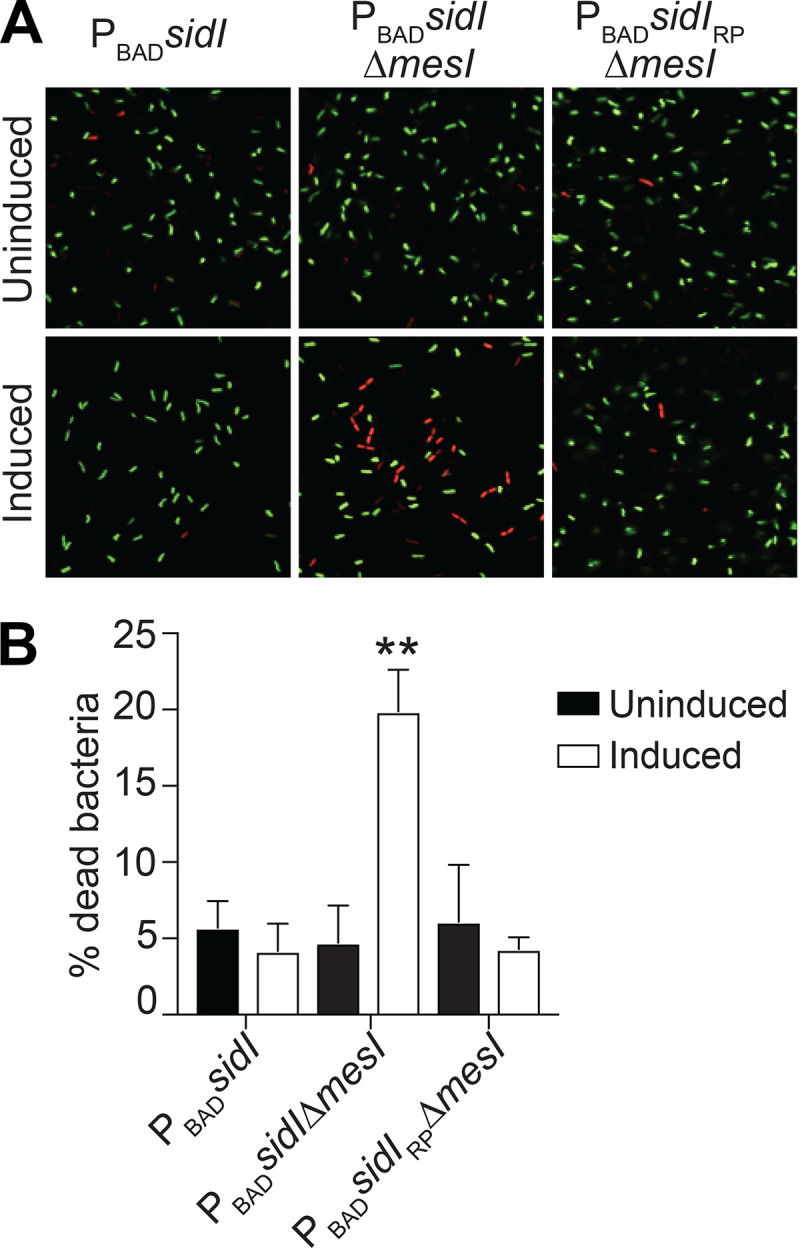
SidI is bactericidal in the absence of MesI. (A) Representative confocal micrographs of Live/Dead-stained L. pneumophila P_BAD_ strains grown in AYE broth under inducing (0.6% arabinose [wt/vol]) or noninducing conditions (SYTO9 [live, green] and propidium iodide [PI] [dead, red]). (B) Quantification of dead L. pneumophila by fluorescence microscopy following Live/Dead staining. Percent dead bacteria was calculated by dividing PI-stained cells by total cells. Data are mean ± SD of 650 cells scored blind, and asterisks denote statistical significance by Student's *t* test (**, *P *< 0.01; compared to P_BAD_*sidI* uninduced). Data are representative of two independent experiments.

SidI impairs L. pneumophila growth, but it is unclear whether SidI is bacteriostatic or bactericidal. SidI is toxic to yeast (11); thus, we tested the hypothesis that SidI is bactericidal when uncoupled from MesI. L. pneumophila P_BAD_ strains (Fig. 2A) were grown for 24 h under arabinose-inducing or noninducing conditions in broth, and viability was evaluated using LIVE/DEAD viability staining and confocal microscopy (Fig. 4). Very few dead bacteria were observed under noninducing conditions or when induced *sidI* expression was coupled with *mesI* (P_BAD_*sidI*) (Fig. 4). However, significantly more dead bacteria were observed when enzymatically active *sidI* was uncoupled from *mesI* under inducing conditions (Fig. 4). We also observed that bacterial growth was rapidly blocked and CFU decreased over time when *sidI* expression was induced in exponentially growing L. pneumophila P_BAD_*sidI*Δ*mesI* strain cultures (see Fig. S1 in the supplemental material). These data suggest that SidI toxicity is conserved across the kingdoms of life and suppressed by MesI.

10.1128/msphere.00552-22.1 FIG S1 Induced expression of *sidI* in exponentially growing MesI-deficient L. pneumophila abolishes bacterial growth *in vitro*. L. pneumophila P_BAD_*sidI*Δ*mesI* was grown in AYE broth for 48 h with the addition of 1% arabinose (wt/vol; induced) or vehicle (sterile water) after 24 h as indicated by the dashed vertical line. CFU were enumerated at the indicated time points by collecting samples from culture tubes and plating on solid media. Data shown are mean ± SD of samples in triplicates, and data shown are representative of three independent experiments. Asterisks denote statistical significance by Student’s *t* test (**, *P *< 0.05). Download FIG S1, PDF file, 0.4 MB. Copyright © 2023 Chauhan et al. 2023 Chauhan et al. https://creativecommons.org/licenses/by/4.0/ This content is distributed under the terms of the Creative Commons Attribution 4.0 International license.

### Intrabacterial regulation of SidI by MesI is sufficient for L. pneumophila virulence.

**FIG 5:**
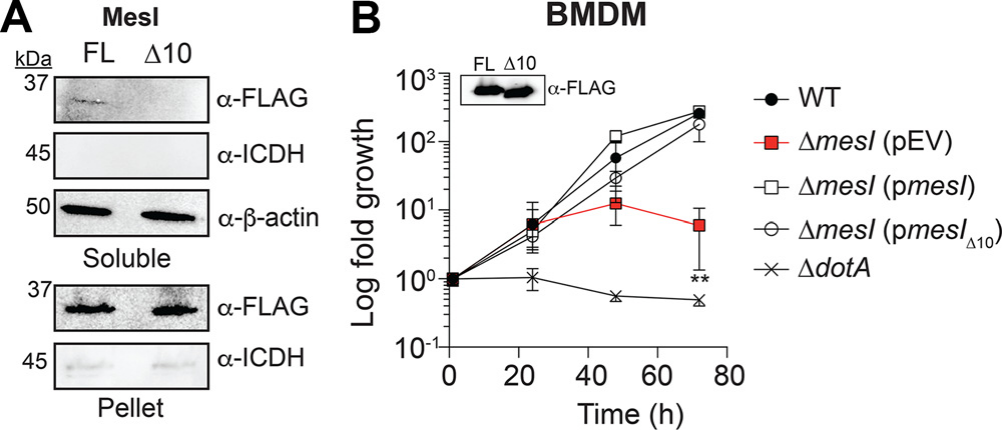
MesI translocation is dispensable for L. pneumophila intracellular replication. (A) Western blot for 3xFLAG-MesI fusion proteins in saponin-solubilized lysates of HEK293 FcγRII cells infected with antibody-opsonized L. pneumophila Δ*mesI* strain harboring p*mesI* (3xFLAG-MesI) or p*mesI*_Δ10_ (3xFLAG-MesIΔ10) at an MOI of 50. Expression of 3xFLAG-MesI fusions was confirmed by Western blotting on saponin-insoluble pellets. ICDH and β-actin were used as controls for bacterial lysis and equal loading, respectively. Data are representative of three independent experiments. (B) Replication of L. pneumophila strains within *Nlrc4*^−/−^ BMDMs infected at an MOI of 1. Fold growth was calculated by normalizing CFU counts to internalized bacteria at 1 h postinfection. Plasmid expression of *mesI* alleles was induced with 1 mM IPTG and confirmed by Western blotting (inset). Asterisks denote statistical significance by Student's *t* test (**, *P *< 0.05) on samples in triplicates. Data are representative of at least three independent experiments.

Based on our observation that SidI is toxic to L. pneumophila when uncoupled from MesI, we hypothesized that MesI intrabacterial regulation of SidI is sufficient for L. pneumophila virulence. We tested this by evaluating whether an intrabacterially retained MesI mutant could complement the Δ*mesI* mutation. We generated an allele of *mesI* lacking 10 amino acids from its extreme carboxy terminus; these amino acids are dispensable for SidI binding and contain the putative E-block Dot/Icm translocation signal (19, 20). We cloned this allele into a complementing plasmid downstream of a *3×flag* epitope tag (p*mesI*_Δ10_) and transformed the plasmid into the *L. pneumophila* ΔmesI strain. We evaluated 3xFLAG-MesIΔ10 translocation using Western blotting to visualize its abundance in saponin-soluble or -insoluble lysate fractions from L. pneumophila-infected cells, a technique routinely used to evaluate effector secretion, since saponin solubilizes eukaryotic membranes but minimally lyses L. pneumophila (21, 22). HEK293 FcγRII cells were infected with antibody-opsonized L. pneumophila harboring either p*mesI* or p*mesI*_Δ10_. 3xFLAG-MesIΔ10 was not translocated since it was retained in the insoluble fraction (pellet) and not present in saponin-soluble lysate fractions (Fig. 5A). As expected, wild-type MesI was secreted into host cells since it was present within saponin-soluble lysate fractions (Fig. 5A). Isocitrate dehydrogenase (ICDH) and β-actin were used as bacterial lysis and loading controls, respectively. We also confirmed that MesIΔ10 interacts with SidI since it was retained by SidI on beads similarly to full-length MesI (see Fig. S2 in the supplemental material).

10.1128/msphere.00552-22.2 FIG S2 MesIΔ10 binds SidI. Glutathione agarose beads were incubated with lysates from E. coli producing GST-SidI or GST (beads) and subsequently incubated with lysates from E. coli producing His_6_-Myc-MesI or -MesIΔ10. Proteins on beads were separated by SDS-PAGE and visualized by Coomassie brilliant blue (CBB) staining or α-Myc Western blotting as indicated. Data are representative of three independent experiments. Download FIG S2, PDF file, 0.2 MB. Copyright © 2023 Chauhan et al. 2023 Chauhan et al. https://creativecommons.org/licenses/by/4.0/ This content is distributed under the terms of the Creative Commons Attribution 4.0 International license.

To evaluate a role for intrabacterial SidI regulation by MesI in L. pneumophila virulence, we tested whether the *mesI*_Δ10_ allele genetically complements Δ*mesI* mutation. We infected BMDMs and quantified replication of wild-type L. pneumophila and Δ*mesI* strains harboring p*mesI*, p*mesI*_Δ10_, or empty plasmid vector (pEV). We were able to genetically complement the Δ*mesI* strain with the *mesI*_Δ10_ allele, demonstrating that MesI translocation into host cells is not necessary for L. pneumophila intracellular replication (Fig. 5B). These data suggest that intrabacterial regulation of SidI by MesI is sufficient for L. pneumophila virulence.

### MesI-deficient bacteria are degraded within host cells.

We found that MesI suppresses intrabacterial SidI toxicity and that MesI translocation into host cells is dispensable for L. pneumophila intracellular replication. Since SidI is also toxic to eukaryotic cells, we initially postulated that the virulence defect associated with loss of MesI results from SidI toxicity in host cells. However, we were unable to detect SidI-mediated cytotoxicity in L. pneumophila-infected BMDMs (see Fig. S3 in the supplemental material), and our new data suggest a potential role for MesI suppression of intrabacterial SidI toxicity in L. pneumophila’s virulence strategy.

10.1128/msphere.00552-22.3 FIG S3 Host cell death is not increased by SidI-producing L. pneumophila. Lactate dehydrogenase activity in supernatants of *Nlrc4*^−/−^ BMDMs infected with the indicated L. pneumophila strains (MOI of 30) for 1, 6, or 10 h. Plasmid expression of *sidI* was induced with 1 mM IPTG. Data are representative of three independent experiments, and statistical analysis was performed using Student’s *t* test (ns, not significant). Download FIG S3, PDF file, 0.2 MB. Copyright © 2023 Chauhan et al. 2023 Chauhan et al. https://creativecommons.org/licenses/by/4.0/ This content is distributed under the terms of the Creative Commons Attribution 4.0 International license.

**FIG 6:**
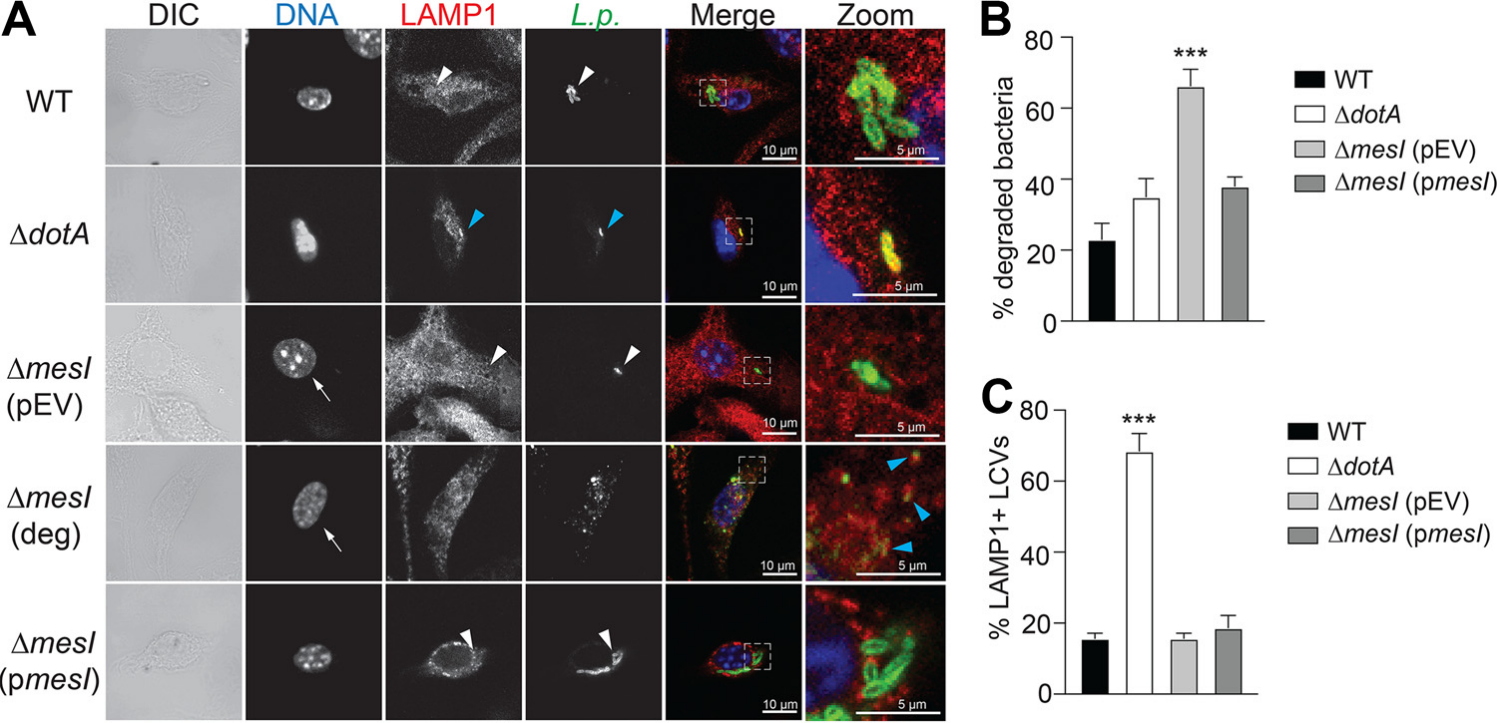
MesI-deficient bacteria are degraded within host cells. (A) Representative confocal micrographs of *Nlrc4*^−/−^ BMDMs infected for 8 h (MOI of 30) with L. pneumophila wild-type (WT), Δ*dotA*, Δ*mesI* (pEV), or Δ*mesI* (p*mesI*) strains and stained with α-L. pneumophila and α-LAMP1 antibodies. Degraded bacteria were identified as α-L. pneumophila (*L.p.*) puncta (see Δ*mesI* [deg]). White arrowheads show in-tact bacteria and LAMP1 association is indicated with blue arrowheads. Nuclei of Δ*mesI* strain-infected BMDMs are indicated with arrows. Quantification of degraded L. pneumophila (B) or LAMP1+ bacteria (C) within *Nlrc4*^−/−^ BMDMs infected for 1 or 8 h at an MOI of 30 in triplicates. Only in-tact bacilli were scored for LAMP1 association. Percent degraded bacteria and LAMP1^+^ bacteria were enumerated by blinded immunofluorescence scoring (*n *= 300). p*mesI* expression was induced with 1 mM IPTG. pEV, empty vector. Data are shown as mean ± SD of samples in triplicates, and asterisks denote statistical significance (***, *P* < 0.01) by one-way ANOVA. Data shown are representative of three independent experiments.

Our data show that SidI is toxic to L. pneumophila and suggest that MesI regulation of SidI within the bacterium is sufficient for intracellular replication. However, our SidI toxicity phenotypes were observed exclusively *in vitro* under inducing conditions, and it is unclear whether intrabacterial SidI toxicity contributes to the Δ*mesI* strain virulence defect. Since dead and dying bacteria are rapidly degraded by host cells, we rationalized that MesI-deficient bacteria would be degraded more robustly within host cells compared to L. pneumophila control strains. Transcriptomic analysis and *in vitro* expression data suggest that *sidI* is constitutively expressed at low levels but that expression increases from 4 to 18 h postinfection and that *sidI* expression is ~3-fold higher in intracellularly growing bacteria compared to broth-grown bacteria (11, 18). Thus, we quantified degraded bacteria by immunofluorescence microscopy and blinded scoring at 8 h postinfection. Degraded L. pneumophila are easily identified using immunofluorescence microscopy since they have lost their rod shape and appear instead as diffuse puncta when cells are stained with a *Legionella*-specific antibody (23, 24). We also evaluated whether MesI-deficient bacteria are able to subvert host endocytic maturation by quantifying association of the lysosomal marker LAMP1 with LCVs (25). We infected BMDMs with the L. pneumophila Δ*mesI* strain and compared degradation and LAMP1 localization to those of virulent L. pneumophila and the L. pneumophila Δ*dotA* strain, which is unable to evade lysosomal fusion (25). Cells were fixed and stained at 8 h postinfection, at which time *sidI* expression has increased and virulent bacteria have begun to replicate in a mature LCV (25, 26). We found that significantly more L. pneumophila Δ*mesI* bacteria were degraded than both wild-type and Δ*dotA* control strains (Fig. 6A and B). It is unlikely that bacterial degradation was accompanied by host cell death since macrophages harboring degraded bacteria had normal nuclear morphology (Fig. 6A, arrows). Furthermore, LCVs harboring in-tact L. pneumophila Δ*mesI* bacilli were largely devoid of LAMP1, similar to L. pneumophila WT and in contrast to the avirulent Δ*dotA* strain (Fig. 6A and C). We did observe LAMP1 staining around degraded bacterial puncta, suggesting that bacteria are degraded in lysosomal compartments (Fig. 6A, Δ*mesI* [deg]; blue arrowheads). Bacterial degradation was significantly decreased when the Δ*mesI* mutation was complemented, and as expected, this strain retained the ability to avoid lysosomal fusion (Fig. 6A and B). The robust degradation of the L. pneumophila Δ*mesI* strain and absence of obvious lysosomal targeting suggests a mechanism of bacterial attenuation distinct from the inability to subvert endocytic maturation.

## DISCUSSION

**FIG 7:**
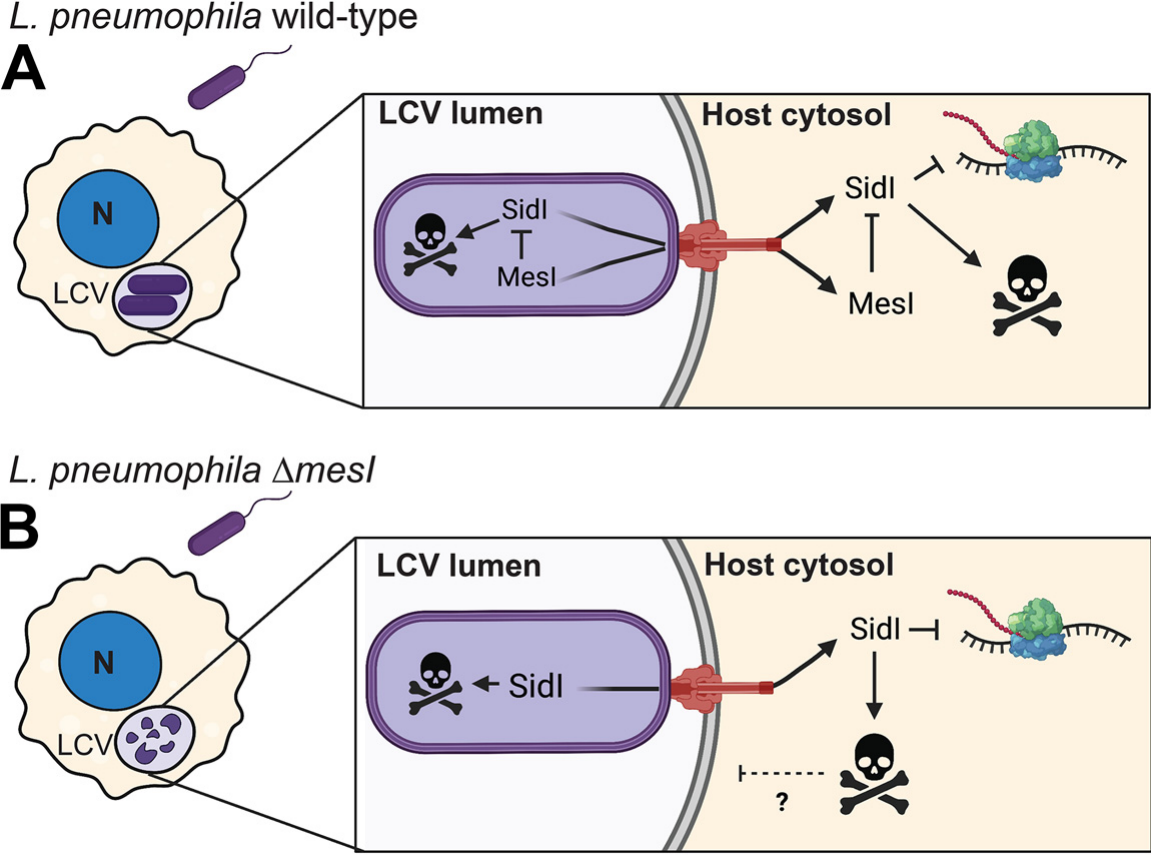
Model for the MesI regulatory mechanism. (A) MesI regulates SidI toxicity within host cells, and the bacterium promotes replication of virulent L. pneumophila (purple) within host cells by regulating SidI within the bacterium and host cell. SidI inhibits eukaryotic protein synthesis and is toxic to both host cells and L. pneumophila in the absence of MesI. (B) MesI-deficient bacteria are degraded within host cells, which is likely a consequence of intrabacterial SidI toxicity. A role for SidI host cell toxicity in the L. pneumophila Δ*mesI* strain virulence defect is unclear (dashed line). Dot/Icm T4SS is shown in red. Figure created in BioRender (https://biorender.com).

This study supports a unique model whereby the L. pneumophila metaeffector MesI drives virulence by intrabacterial regulation of its cognate effector SidI (Fig. 7). Several L. pneumophila effectors are toxic to eukaryotic cells; however, effector bactericidal activity in L. pneumophila has not been previously observed. Furthermore, our data challenge the dogma that metaeffectors promote L. pneumophila virulence by functioning exclusively within host cells. SidI and MesI bear resemblance to canonical toxin-antitoxin (TA) and effector-immunity (E-I) pairs (27–29); however, they are distinct since antitoxin/immunity proteins, by definition, are not secreted (30). This also distinguishes MesI from canonical chaperones, which remain intrabacterial (31). Since MesI is a translocated substrate of the Dot/Icm T4SS, regulation of SidI within the host cell is also likely important for L. pneumophila virulence. Current data show that *sidI* expression is upregulated during the exponential growth phase and that expression of *mesI* is temporally delayed relative to *sidI* (18, 19). These data suggest that MesI, like other metaeffectors, temporally regulates its cognate effector (8). We postulate that the temporal delay in *mesI* expression allows the bacterium to both “turn off” SidI activity in the host cell and prevent intrinsic collateral damage. Our data suggest that intrabacterial MesI activity drives L. pneumophila virulence in laboratory infection models; however, it is also important to define the role and mechanism by which Dot/Icm-translocated MesI functions within the host cell cytosol.

Our data suggest that MesI promotes L. pneumophila virulence by suppressing intrabacterial SidI toxicity and challenge the initial assumption that impaired replication was due to SidI-mediated host cell death. We found that Dot/Icm-translocated SidI did not increase host cell death and that macrophages harboring both in-tact and degraded L. pneumophila Δ*mesI* had normal morphology. Moreover, our data suggest that L. pneumophila Δ*mesI* strains are killed prior to degradation in host lysosomes since very few of the remaining in-tact bacilli localized with LAMP1. SidI activity is dose dependent, and we think that LCV biogenesis occurs normally until *sidI* expression increases after ~6 h of growth (11, 18). It is very likely that viable L. pneumophila Δ*mesI* strains devoid of LAMP1 at 8 h postinfection may have genomic variations that suppress SidI toxicity. Further investigation is required to fully define the kinetics of LCV biogenesis and bacterial degradation when SidI is uncoupled from MesI.

Interkingdom SidI toxicity suggests that its target is highly conserved between L. pneumophila and host cells. SidI is one of at least seven L. pneumophila effectors that subvert host protein synthesis (11, 32), one of the most conserved cellular processes across the kingdoms of life (33). Effector-mediated suppression of host mRNA translation is important for L. pneumophila’s acquisition of host-derived amino acids, which are essential for intracellular replication and the main driver of bacterial phase switching within host cells (23, 34). The mechanism by which SidI inhibits protein synthesis is unclear; however, SidI binds eukaryotic elongation factor 1A (eEF1A) and likely functions as a mannosyltransferase, so it is tempting to speculate that SidI blocks translation by modifying eEF1A (11, 12). However, the functional significance of SidI-eEF1A binding remains unclear since it is unaffected by MesI and insufficient for translation inhibition (11, 12). L. pneumophila has evolved strategies to prevent intrabacterial suppression of translation. For example, the effector Lgt1 blocks eukaryotic translation by glycosylating eEF1A on Ser53, which is conserved in eukaryotes but not the bacterial eEF1A homolog, EF-Tu (35, 36). The mechanistic underpinnings of intrabacterial MesI activity is unclear; however, it is tempting to speculate that MesI may function in part to preserve protein synthesis within the bacterium.

The mechanism of intrabacterial SidI toxicity is unknown. SidI cleaves GDP-mannose and has predicted structural homology to mannosyltransferase enzymes (12). We think that SidI toxicity results from mannosylation of translation factors conserved between the host and L. pneumophila. However, hydrolysis of GDP-mannose may have other deleterious effects within the bacterium. For example, mannose comprises 4% of the core oligosaccharide in L. pneumophila lipopolysaccharide (LPS), and depletion of GDP-mannose by excess SidI activity may adversely affect cell envelope biosynthesis as has been suggested for MutT family enzymes in Escherichia coli (37, 38). Interestingly, SidI shares predicted structural homology with phosphatidyl-myo-inositol mannosyltransferases that contribute to cell envelope biosynthesis in mycolated pathogens, including *Mycobacterium* and *Corynebacterium* spp. (12, 39, 40). The biological relevance of this activity in L. pneumophila, which does not have a mycolated cell envelope, is unclear. However, whether impaired LPS biosynthesis contributes to SidI intrabacterial toxicity is unknown and warrants investigation.

Recent work has challenged the dogma that bacterial effectors function exclusively within host cells. Hardwidge and colleagues revealed that the pathogenic E. coli effector NleB and Salmonella enterica serovar Typhimurium effector SseK1 function intrabacterially. NleB confers resistance to oxidative stress and SseK1 confers resistance to methylglyoxal and regulates UDP-GlcNAc biosynthesis (41–43). The pathogenic E. coli effector NleC can also function intrabacterially, but the endogenous, biologically relevant substrates are unknown (44). Our data suggest that intrabacterial MesI activity confers a fitness advantage on L. pneumophila by suppressing intrabacterial SidI activity. Interestingly, SidI appears to be constitutively produced at low levels when L. pneumophila is grown in broth (19), suggesting that low levels of SidI activity within the bacterium may be beneficial at early stages of the L. pneumophila life cycle. Indeed, there is emerging evidence that bacterial TA pairs can shape pathogen physiology by modulating posttranscriptional gene expression (45). The Escherichia coli toxin RelE potently blocks translation and is neutralized by the antitoxin RelB when the bacteria are grown in nutrient rich environments (46–48). However, under limiting conditions, RelE activity contributes to adaptation to nutrient starvation by suppressing bacterial translation, and when nutrients are restored, RelB production increases and translation resumes (48). Since L. pneumophila requires exogenous amino acids to replicate, it is tempting to speculate that SidI may suppress translation until its replicative niche within the host cell is established. Thus, SidI and MesI may be an ancient TA pair that have been co-opted by the T4SS to modulate both host and pathogen physiology.

Our data suggest that intrabacterial SidI toxicity is responsible for impaired L. pneumophila Δ*mesI* strain intracellular replication. However, McCloskey et al. recently proposed a model whereby MesI negatively regulates SidI translocation into host cells (19). MesI binds the extreme C terminus of SidI, which encodes the canonical E-block Dot/Icm translocation signal (12, 19, 49) however, the authors found no differences in SidI secretion when the relative abundance of MesI was increased (19). Furthermore, we did not detect any MesI-mediated differences in SidI translocation from L. pneumophila using an established adenylate cyclase (CyaA) reporter (12). This discrepancy may be a consequence of differences in experimental conditions. Further investigation is required to define the impact of MesI on SidI translocation and the potential role of this activity in the MesI regulatory mechanism and L. pneumophila virulence.

Together, this study revealed a unique role for intrabacterial regulation of a translocated effector by its cognate metaeffector in L. pneumophila virulence. Our data, in the context of previously published studies (11, 12), suggest that SidI’s target is conserved between host and pathogen. Recent phylogenetic analysis suggests ancient and extensive coevolution between the order *Legionellales*, which includes *Legionella* spp., and unicellular eukaryotes (50). Thus, metaeffectors may represent an ancient mechanism evolved by pathogenic bacteria for adaptation to eukaryotic hosts.

## MATERIALS AND METHODS

### Bacterial strains, plasmids, primers, and culture conditions.

Escherichia coli Top10 (cloning), DH5αλ*pir* (allelic exchange), and BL21(DE3) (protein production) were maintained at 37°C in Luria-Bertani (LB) medium supplemented with appropriate antibiotics for plasmid maintenance (50 μg mL^−1^ kanamycin, 25 μg mL^−1^ chloramphenicol, or 100 μg mL^−1^ ampicillin). Protein production in E. coli BL21(DE3) was induced with 1 mM isopropyl-β-d-1-thiogalactopyranoside (IPTG).

All Legionella pneumophila strains in this study were derived from L. pneumophila Philadelphia-1 SRS43 (10) and cultured at 37°C on charcoal-N (2-acetamido)-2-aminoethanesulfonic acid (ACES)-buffered yeast extract (CYE) supplemented with l-cysteine and ferric pyrophosphate as described previously (51). Single colonies were isolated after 4 days of growth on CYE agar and used to generate 48-h heavy patches of bacteria. Where indicated, liquid cultures were grown from heavy patched bacteria at 37°C in l-cysteine- and ferric pyrophosphate-supplemented ACES-buffered yeast extract broth (AYE) (52). Media were supplemented with 10 μg mL^−1^ chloramphenicol (plasmid maintenance), 10 μg mL^−1^ kanamycin (allelic exchange), 1 mM IPTG (P*tac* gene expression), or 0.6 to 2% L-arabinose (wt/vol) (P_BAD_ gene expression).

Plasmids and oligonucleotide primers used in this study are listed in Table 1 and Table 2, respectively.

**TABLE 1:** Plasmids used in this study

Name and function	Description	Resistance	Reference/source
Protein production			
pT7HMT	Production of His_6_-Myc fusions	Kan^r^	55
pT7HMT::*mesI*	Production of His_6_-Myc-MesI	Kan^r^	12
pT7HMT::*mesI*_Δ10_	Production of His_6_-Myc-MesIΔ10	Kan^r^	This study
pGEX6P1	Production of GST	Amp^r^	GE healthcare
pGEX6P1::*sidI*	Production of GST-SidI	Amp^r^	12
Allelic exchange			
pSR47s	Allelic exchange vector	Kan^r^	56
pSR47s::P_BAD_	For chromosomal integration of P_BAD_-*3×flag* upstream of *sidI*	Kan^r^	This study
pSR47s::Δ*dotA*	For in-frame deletion of *dotA*	Kan^r^	This study
Genetic complementation and cloning			
pJB1806	*Legionella* expression vector	Cm^r^	53
pJB1806::*sidI* (p*sidI*)	*sidI* expression from P*tac*	Cm^r^	This study
pJB1806::*sidI*_R453P_ (p*sidI*_RP_)	*sidI*_R453P_ expression from P*tac*	Cm^r^	This study
pSN85	*Legionella* vector with in-frame N-terminal *3×flag* epitope tag	Cm^r^	54
pSN85::*sidI*	To generate *3×flag-sidI*	Cm^r^	This study
pSN85::*mesI* (p*mesI*)	*3×flag*-*mesI* expression from P*tac*	Cm^r^	This study
pSN85::*mesI*_Δ10_ (p*mesI*_Δ10_)	*3×flag*-*mesI*_Δ10_ expression from P*tac*	Cm^r^	This study

**TABLE 2:** Oligonucleotide primers used in this study

Name and function	Sequence^*a*^
Protein production	
MesIT7SalI-F	ATT**GTCGAC**AATGATAAAAGGAAAACTTATGCCC
MesIΔ10NotI-R	ATT**GCGGCCGC**TTACTCATCGTCTTTACC
Allelic exchange	
DotAKOSacI-F	ATT**GTCGACG**CCCTAGCTGTTTCATTGC
DotAKONotI-R	ATT**GCGGCCGCA**CATTCTTTCTCACCCAATAAC
DotAKONotI-F	ATT**GCGGCCGC**A**TAA**CATTCAAAAACAATATA
DotAKOSalI-R	ATT**GAGCTC**CCAAAACAAAATAACCAGCTG
SidIUTR-F	CCCCC**GAGCTC**GAGCATCAATAATATCCTGC
SidIUTR-R	GTAGCCGTCAAGTTGTCAAAATTACTTTAGTAAAATTTTTGTCATGGTG
araC-pBAD-F	CACCATGACAAAAATTTTACTAAAGTAATTTTGACAACTTGACGGCTACATC
araC-pBAD-R	CATGGTCTTTGTAGTCCATATATCTCCTTCTTAAAGTTAAACAAAATTATTTC
3FlagSidI-F	GTTTAACTTTAAGAAGGAGATATATGGACTACAAAGACCATGACG
3FlagSidI-R	CCCCC**GTCGAC**TGACTACACCCCTCTTTATTGGC
Genetic complementation	
SidIBamHI-F	TTA**GGATCC**TTATGACTAAAATATACTTATTAAC
SidISphI-R	ATT**GCATGC**TCAATCACAAATTAATTTTTGCACATC
MesIBglII-F	TGG**AGATCT**TTATGATAAAAGGAAAACTTATGC
MesISphI-R	ATT**GCATGC**TTATAAAATAATTGGTCGAG
MesIΔ10SphI-R	ATT**GCATGC**TTACTCATCGTCTTTACCAGC
qRT-PCR	
sidIRT-F	ATCAGGCAAACCGACGTGAC
sidIRT-R	CCGCAATCTGGTGGACGAAT
16SRT-F	TCAGTGGGGAGGAGGGTTGA
16SRT-R	CGCTCGCACCCTCCGTATTA
Site-directed mutagenesis	
SidIR453P-sense	CCGCAATCTGGTGGAGGAATATGAATATCAAGATGAGTA
SidIR453P-asense	TACTCATCTTGATATTCATATTCCTCCACCAGATTGCGG

a Restriction endonuclease cleavage sites are bolded.

### Molecular cloning and strain construction.

L. pneumophila genomic DNA (gDNA) was isolated using the Illustra genomicPrep DNA spin kit (GE Healthcare) and used as a template for cloning *sidI* and *mesI* into the indicated plasmid vectors. *sidI* was amplified from L. pneumophila gDNA using SidIBamHI-F/SidISphI-R, which includes 60 base pairs (bp) downstream of the *sidI* open reading frame, and cloned as a BamHI/SphI fragment into pJB1806 (53) or downstream of an in-frame *3×flag* epitope tag in pSN85 (54) to generate pJB1806::*sidI* (p*sidI*) and pSN85::*sidI*. To generate p*sidI*_R453P_, p*sidI* was mutagenized using site-directed mutagenesis with primer pairs SidIR453P-sense/SidIR453P-asense (10). To generate p*mesI* complementation plasmids, *mesI* and *mesI*_Δ10_ open reading frames were amplified from L. pneumophila gDNA using MesIBglII-F/MesISphI-R or MesIBglII-F/MesIΔ10SphI-R primer pairs, respectively, and cloned into pSN85 in-frame with the *3×flag* epitope tag. Ligations were transformed into chemically competent E. coli Top10 and sequences confirmed by Sanger sequencing (Eton Biosciences). L. pneumophila complementation strains were generated by electroporation of plasmids into competent L. pneumophila strains using a Bio-Rad Gene Pulser at 2.4 kV, 200 Ω, and 0.25 μF and plated on CYE supplemented with 10 μg mL^−1^ chloramphenicol.

For production of recombinant His_6_-Myc-MesIΔ10, *mesI*Δ10 was amplified from L. pneumophila gDNA using MesIT7SalI-F/MesIΔ10NotI-R and cloned as a SalI/NotI fragment into pT7HMT (55) downstream of an in-frame His_6_-Myc epitope tag. pT7HMT::*mesI* and pGEX::*sidI* were generated previously (Table 2) (12). Ligation reactions were transformed into chemically competent E. coli Top10, and sequences were confirmed by Sanger sequencing (Eton Biosciences).

The *dotA* open reading was deleted from the L. pneumophila chromosome using allelic exchange as described (56). To generate a clean in-frame deletion of *dotA*, 5′ and 3′ regions flanking the *dotA* open reading frame (726 bp upstream and 1,012 bp downstream) were amplified using DotAKOSacI-F/DotAKONotI-R and DotAKONotI-F/DotAKOSalI-R to generate SacI/NotI and NotI/SalI fragments, which were ligated into SacI/SalI digested pSR47s to generate pSR47s::Δ*dotA*, which was conjugated into L. pneumophila SRS43 for selection of double crossover events, as described. Sequences were confirmed by Sanger sequencing (Eton Biosciences) and the Δ*dotA* phenotype was confirmed by comparison with the established SRS43 *dotA*::Tn strain (10).

For allelic exchange to insert the *araC*-*araBAD* (P_BAD_) promoter and a *3×flag* epitope tag upstream of *sidI* in the L. pneumophila chromosome, overlapping primers were used to generate a fusion construct consisting of 1,000 bp upstream of *sidI* (SidIUTR-F/SidIUTR-R; from L. pneumophila gDNA), P_BAD_ promoter (araC-pBAD-F/araC-pBAD-R; from pMRBAD::z-CspGFP [a gift from Brian McNaughton; Addgene number 40730] [57]), and *3×flag* fused to the first 1,200 nucleotides of *sidI* (3FLAG-SidI-F/3FLAG-SidI-R; from pSN85::*sidI*), which was ligated as a SacI/SalI fragment into pSR47s to generate pSR47S::P_BAD_. Plasmids were transformed into chemically competent E. coli DH5αλ*pir*, and sequences were confirmed by Sanger sequencing (Eton Biosciences). To generate P_BAD_ strains, pSR47s::P_BAD_ was conjugated into the L. pneumophila SRS43 wild-type, Δ*mesI*, and *sidI*_R453P_Δ*mesI* strains (10) and selection of double crossover events was performed as described (56). Sucrose resistant, kanamycin-sensitive colonies were screened by PCR to verify gene insertion.

### Mice.

C57BL/6 *Nlrc4*^−/−^ mice (a gift from Craig Roy) have been described (58). In-house colonies were maintained under specific pathogen-free conditions at Kansas State University. Bone marrow was harvested from 8- to 15-week-old mice as previously described (59). All experiments involving animals were approved by the Kansas State University Institutional Animal Care and Use Committee (IACUC-4501 and -4022) and performed in compliance with the Animal Welfare Act and National Institutes of Health guidelines.

### Cell culture.

All mammalian cells were grown at 37°C/5% CO_2_. HEK293 FcγRII cells (a gift from Craig Roy) were cultured in Dulbecco's modified Eagle medium (DMEM) (Gibco) supplemented with 10% heat-inactivated fetal bovine serum (HIFBS) (Biowest) for up to 25 passages. Primary mouse bone marrow-derived macrophages (BMDMs) were differentiated in RPMI supplemented with 20% HIFBS (Biowest) and 15% L-929 conditioned medium (Differentiation media) for 6 days prior to infection as described (59). Differentiated BMDMs were seeded for infections in RMPI supplemented with 10% HIFBS and 7.5% L-929 conditioned medium (seeding medium).

### Macrophage growth curves.

BMDMs were seeded into 24-well tissue culture dishes at 2.5 × 10^5^ per well 1 day prior to infection. L. pneumophila were cultured on CYE agar and heavy patch-grown bacteria were used to infect BMDMs at an multiplicity of infection (MOI) of 1 in triplicates, and CFU were enumerated as previously described (10, 15). Fold growth was calculated by normalizing CFU at 24 h, 48 h, and 72 h to internalized bacteria at 1 h postinfection. For genetic complementation, either 1 mM IPTG or 2% l-arabinose (wt/vol) was added to the medium as indicated at the time of infection and maintained throughout.

### *In vitro* growth curves.

L. pneumophila heavy patches were resuspended in fresh AYE broth and subcultured for consistent 600 nm optical density (OD_600_). Cultures were split into triplicate wells of a 96-well round-bottom plate and incubated at 37°C with continuous orbital shaking using an Agilent BioTek Epoch2 plate reader. OD_600_ from triplicate wells was read every 2 h for 36 h. Plasmids were maintained with 10 μg mL^−1^ chloramphenicol and IPTG (1 mM) or 1% l-arabinose (wt/vol) were added to the media to induce gene expression as indicated.

To quantify bacterial growth by CFU assay, L. pneumophila heavy patches were resuspended in fresh AYE broth and subcultured (OD_600_ = 0.2), and 3 mL was added to each of six culture tubes. Bacteria were grown at 37°C with shaking. After 24 h, *sidI* expression was induced in three culture tubes with 1% l-arabinose ([vol/vol] from 30% [wt/vol] stock), a volume equivalent of sterile water (vehicle) was added to the other three culture tubes, and bacteria were grown for an additional 24 h. Samples were taken from culture tubes for plating and CFU enumeration after 3, 6, 10, 24, 27, 30, 34, and 48 h of growth.

### Quantitative RT-PCR.

L. pneumophila strains were grown for 1 or 6 h in AYE broth of incubation with and without arabinose (2%), and total RNA was purified with the Direct-zol RNA miniprep kit with TRI reagent (Zymo Research) following the manufacturer’s instructions. RNA samples were treated with DNase (Sigma) before reverse transcription with SuperScript III (Invitrogen). Quantitative reverse transcriptase PCR (RT-PCR) was performed using the Invitrogen SuperScript III Platinum SYBR green one-step qRT-PCR kit. Transcript abundance was quantified using sidIRT-F/sidIRT-R (sidI) and 16SRT-F/16SRT-R (16S rRNA) primer pairs on a Bio-Rad CFX96 real-time PCR machine. Fold expression (2^ΔΔ^*^CT^*) was calculated by normalizing *sidI* transcript abundance to 16S rRNA and standardizing values to wild-type L. pneumophila.

### Bacterial viability and LIVE/DEAD staining.

L. pneumophila P_BAD_ strains were resuspended in AYE media from a heavy patch (48 h). Strains were grown for 24 h at 37°C in the presence or absence of 0.6% l-arabinose (wt/vol). Cell viability was quantified using a LIVE/DEAD *Bac*Light bacterial viability kit according to manufacturer’s instructions. Bacteria were stained with 5 μM SYTO 9 (live; green) and 10 μM propidium iodide (dead; red) (Thermo Fisher) and incubated for 15 min at room temperature in the dark. To determine the baseline threshold for dead cells, a negative control was used, where cells were treated with 90% ethanol for 1 h. After staining, cells were washed with sterile water, and 5 μL of resuspended cells were loaded on a glass slide with a coverslip. Images were acquired at the KSU Division of Biology Microscopy Facility using a Zeiss LSM5 laser scanning confocal microscope using a 100× oil-immersion objective. Percent dead bacteria was calculated by normalizing dead bacteria (red) to total bacteria using ImageJ software. At least 500 cells were evaluated for each strain and culture condition, and the researcher performing the analysis was blinded to sample identity.

### Western blotting.

Protein was boiled in Laemmli buffer, separated by SDS-PAGE, and transferred to polyvinylidene difluoride (PVDF) (Fisher Scientific) membranes using a Bio-Rad wet transfer apparatus. The membranes were incubated with blocking buffer (5% nonfat milk powder dissolved in Tris-buffered saline-0.1% Tween 20 [TBST]) for 30 min. Membranes were incubated with primary antibodies (mouse α-FLAG M2 [1:1,000; Sigma]; rabbit α-ICDH [1:1,000; Sigma]; rabbit α-β-actin [Cell Signaling]; rabbit α-Myc TAG MAb [71D10] [1:1,000; Cell Signaling]) at 1:1,000 in blocking buffer overnight at 4°C with rocking and subsequently with horseradish peroxidase (HRP)-conjugated goat α-rabbit or α-mouse secondary antibodies at 1:5,000 (Thermo Fisher) in blocking buffer for 2 h at room temperature with rocking. Membranes were washed, incubated with ECL reagent (GE Amersham), and imaged by chemiluminescence using an Azure Biosystems c300 Darkroom Replacer.

### Effector secretion assay.

HEK293 FcγRII cells (60) were seeded in 10-cm poly-l-lysine-coated tissue culture dishes (4 × 10^6^) 1 day prior to infection. L. pneumophila strains were patched from fresh single colonies onto CYE agar supplemented with 10 μg mL^−1^ chloramphenicol and 1 mM IPTG to induce gene expression. Bacteria were opsonized with α-L. pneumophila antibody (1:1,000 [Invitrogen; PA17227]) in DMEM 10% HIFBS supplemented with 1 mM IPTG for 20 min at room temperature (RT) with rotation. Cells were infected with opsonized bacteria at an MOI of 50 in for 2 h in 2 mL DMEM 10% HIFBS supplemented with 1 mM IPTG. Cells were washed 3× with ice-cold phosphate-buffered saline (PBS) to remove extracellular bacteria and lysed for 10 min in 500 μL ice-cold Hank’s balanced salt solution (HBSS) supplemented with 0.2% saponin and ProBlock gold mammalian protease inhibitor cocktail (GoldBio). Lysates were treated with RNase A (10 μg mL^−1^) and DNase I (10 μg mL^−1^), incubated at RT for 15 min, and centrifuged for 15 min at 17,000 relative centrifugal force (rcf) at 4°C. Saponin-soluble supernatants were filtered using a 0.22 μM syringe filter and transferred to a fresh 1.5-mL microcentrifuge tube. Saponin-insoluble pellets were resuspended in 50 μL TE buffer (10 mM Tris-HCl, 1 mM EDTA). Fifty microliters of supernatant and pellet fractions were diluted in 3× Laemmli sample buffer and boiled for 10 min, and the whole sample was loaded into wells of a 1.5-mm 15% SDS-PAGE gel for Western blotting.

### Affinity chromatography.

Affinity chromatography from E. coli lysates was performed as described (12). Briefly, E. coli BL21(DE3) harboring pT7HMT::*mesI*, pT7HMT::*mesI*_Δ10_, pGEX6P1::*sidI*, or pGEX6P1 were grown overnight with shaking at 37°C and subcultured at 1:100 in LB media. Subcultures were grown for 3 h followed by induction with 1 mM IPTG and growth overnight at 16°C. Clarified lysates from bacteria producing glutathione *S*-transferase (GST) fusion proteins were incubated with pre-equilibrated glutathione magnetic agarose beads (Pierce) for 1 h at 4°C with rotation. Beads were washed twice in washing buffer (125 mM Tris-Cl, 150 mM NaCl, 1 mM dithiothreitol [DTT], 1 mM EDTA, pH 7.4) and incubated with clarified lysates from bacteria producing His_6_-Myc fusion proteins at 4°C for 1 h with rotation. Beads were washed twice in washing buffer transferred to a fresh 1.5-mL microcentrifuge tube and boiled in 25 μM 3× Laemmli sample buffer. Proteins were separated by SDS-PAGE and visualized using Coomassie brilliant blue or Western blotting as indicated.

### Cytotoxicity assay.

Cytotoxicity was evaluated by quantifying lactate dehydrogenase (LDH) activity in supernatants of L. pneumophila-infected BMDMs. BMDMs were seeded in 24-well tissue culture dishes at 2.5 × 10^5^ in seeding medium 1 day prior to infection. Cells were infected with L. pneumophila strains at an MOI of 50 for 1 h, washed 3 times with sterile phosphate-buffered saline (PBS^−/−^), and incubated in seeding media for additional 5 h or 9 h. At the indicated times, plates were centrifuged at 250 rcf, and supernatants were transferred to a sterile non-tissue-culture-treated 96-well plate. For the 1 h time point, supernatants were collected from cells without washing. LDH was quantified using the CytoTox 96 nonradioactive cytotoxicity assay (Promega) according to manufacturer’s instructions. Absorbance at 490 nm was read on a BioTek Epoch2 microplate reader, and percent cytotoxicity was calculated by normalizing absorbance values to cells treated with lysis buffer.

### Immunofluorescence microscopy and scoring.

BMDMs (1 × 10^5^) were seeded on poly-l-lysine-coated glass coverslips in 24-well tissue culture dishes 1 day prior to infection. BMDMs were infected in triplicate wells with the indicated L. pneumophila strains at an MOI of 30 for 1 h or 8 h. For the 8-h time point, extracellular bacteria were removed after 1 h by washing coverslips three times in PBS^−/−^ and incubated with fresh media for 7 h. Coverslips were fixed in 4% paraformaldehyde for 15 min and permeabilized with ice-cold methanol. Coverslips were stained with 1:1,000 rabbit α-L. pneumophila primary antibody (Invitrogen; PA17227) and 1:500 Alexa488-conjugated goat α-rabbit secondary antibody (Thermo Fisher). Where indicated, BMDMs were also stained with 1:1,000 rat α-LAMP1 antibody (ID4B; Developmental Studies Hybridoma Bank) and 1:500 Alexa594-conjugated goat α-rat secondary antibody (Thermo Fisher). Nuclei were stained with 1:10,000 Hoechst (Thermo Fisher), and coverslips were mounted on slides with ProLong gold antifade mountant (Invitrogen). Degraded bacteria and LAMP1^+^ LCVs harboring in-tact bacilli were scored blind on a Leica DMiL LED inverted epifluorescence microscope (*n *= 300). Representative images were acquired at the KSU College of Veterinary Medicine Confocal Core using a Zeiss LSM 880 inverted confocal microscope and processed using Fiji ImageJ and Adobe Photoshop software.

### Statistical analysis.

Statistical analysis was performed with GraphPad Prism 9 software using an unpaired two-tailed *t* test with a 95% confidence interval. Unless otherwise indicated, data are presented as mean ± standard deviation (SD), and statistical analysis was performed on triplicate biological replicates.

## References

[1] Horwitz MA, Silverstein SC. 1980. Legionnaires’ disease bacterium (*Legionella pneumophila*) multiples intracellularly in human monocytes. J Clin Invest 66:441–450. doi:10.1172/JCI109874. 7190579 PMC371671

[2] Park JM, Ghosh S, O'Connor TJ. 2020. Combinatorial selection in amoebal hosts drives the evolution of the human pathogen *Legionella pneumophila*. Nat Microbiol 5:599–609. doi:10.1038/s41564-019-0663-7. 31988381 PMC10234074

[3] Gomez-Valero L, Rusniok C, Carson D, Mondino S, Pérez-Cobas AE, Rolando M, Pasricha S, Reuter S, Demirtas J, Crumbach J, Descorps-Declere S, Hartland EL, Jarraud S, Dougan G, Schroeder GN, Frankel G, Buchrieser C. 2019. More than 18,000 effectors in the *Legionella* genus genome provide multiple, independent combinations for replication in human cells. Proc Natl Acad Sci USA 116:2265–2273. doi:10.1073/pnas.1808016116. 30659146 PMC6369783

[4] Swanson MS, Isberg RR. 1995. Association of *Legionella pneumophila* with the macrophage endoplasmic reticulum. Infect Immun 63:3609–3620. doi:10.1128/iai.63.9.3609-3620.1995. 7642298 PMC173501

[5] Horwitz MA. 1983. Formation of a novel phagosome by the Legionnaires’ disease bacterium (*Legionella pneumophila*) in human monocytes. J Exp Med 158:1319–1331. doi:10.1084/jem.158.4.1319. 6619736 PMC2187375

[6] Chauhan D, Shames SR. 2021. Pathogenicity and virulence of *Legionella*: intracellular replication and host response. Virulence 12:1122–1144. doi:10.1080/21505594.2021.1903199. 33843434 PMC8043192

[7] Berger KH, Isberg RR. 1993. Two distinct defects in intracellular growth complemented by a single genetic locus in *Legionella pneumophila*. Mol Microbiol 7:7–19. doi:10.1111/j.1365-2958.1993.tb01092.x. 8382332

[8] Joseph AM, Shames SR. 2021. Affecting the effectors: regulation of *Legionella pneumophila* effector function by metaeffectors. Pathogens 10:108. doi:10.3390/pathogens10020108. 33499048 PMC7911563

[9] Kubori T, Shinzawa N, Kanuka H, Nagai H. 2010. *Legionella* metaeffector exploits host proteasome to temporally regulate cognate effector. PLoS Pathog 6:e1001216. doi:10.1371/journal.ppat.1001216. 21151961 PMC2996335

[10] Shames SR, Liu L, Havey JC, Schofield WB, Goodman AL, Roy CR. 2017. Multiple *Legionella pneumophila* effector virulence phenotypes revealed through high-throughput analysis of targeted mutant libraries. Proc National Acad Sci 114:E10446–E10454. doi:10.1073/pnas.1708553114. PMC5715750 29133401

[11] Shen X, Banga S, Liu Y, Xu L, Gao P, Shamovsky I, Nudler E, Luo Z. 2009. Targeting eEF1A by a *Legionella pneumophila* effector leads to inhibition of protein synthesis and induction of host stress response. Cell Microbiol 11:911–926. doi:10.1111/j.1462-5822.2009.01301.x. 19386084 PMC2967282

[12] Joseph AM, Pohl AE, Ball TJ, Abram TG, Johnson DK, Geisbrecht BV, Shames SR. 2020. The *Legionella pneumophila* metaeffector Lpg2505 (MesI) regulates SidI-mediated translation inhibition and novel glycosyl hydrolase activity. Infect Immun 88:e00853-19. doi:10.1128/IAI.00853-19. 32122942 PMC7171249

[13] Ren T, Zamboni DS, Roy CR, Dietrich WF, Vance RE. 2006. Flagellin-deficient *Legionella* mutants evade caspase-1- and Naip5-mediated macrophage immunity. PLoS Pathog 2:e18. doi:10.1371/journal.ppat.0020018. 16552444 PMC1401497

[14] Molofsky AB, Byrne BG, Whitfield NN, Madigan CA, Fuse ET, Tateda K, Swanson MS. 2006. Cytosolic recognition of flagellin by mouse macrophages restricts *Legionella pneumophila* infection. J Exp Med 203:1093–1104. doi:10.1084/jem.20051659. 16606669 PMC1584282

[15] Ngwaga T, Hydock AJ, Ganesan S, Shames SR. 2019. Potentiation of cytokine-mediated restriction of *Legionella* intracellular replication by a Dot/Icm-translocated effector. J Bacteriol 201:e00755-18. doi:10.1128/JB.00755-18. 31036725 PMC6597390

[16] Falkow S. 2004. Molecular Koch’s postulates applied to bacterial pathogenicity — a personal recollection 15 years later. Nat Rev Microbiol 2:67–72. doi:10.1038/nrmicro799. 15035010

[17] Falkow S. 1988. Molecular Koch’s postulates applied to microbial pathogenicity. Rev Infect Dis 10:S274–S276. doi:10.1093/cid/10.supplement_2.s274. 3055197

[18] Faucher SP, Mueller CA, Shuman HA. 2011. *Legionella pneumophila* transcriptome during intracellular multiplication in human macrophages. Front Microbiol 2:60. doi:10.3389/fmicb.2011.00060. 21747786 PMC3128937

[19] McCloskey A, Perri K, Chen T, Han A, Luo Z-Q. 2021. The metaeffector MesI regulates the activity of the *Legionella* effector SidI through direct protein-protein interactions. Microbes Infect 23:104794. doi:10.1016/j.micinf.2021.104794. 33571674 PMC9406241

[20] Burstein D, Zusman T, Degtyar E, Viner R, Segal G, Pupko T. 2009. Genome-scale identification of *Legionella pneumophila* effectors using a machine learning approach. PLoS Pathog 5:e1000508. doi:10.1371/journal.ppat.1000508. 19593377 PMC2701608

[21] Puvar K, Iyer S, Fu J, Kenny S, Terón KIN, Luo Z-Q, Brzovic PS, Klevit RE, Das C. 2020. Legionella effector MavC targets the Ube2N~Ub conjugate for noncanonical ubiquitination. Nat Commun 11:2365. doi:10.1038/s41467-020-16211-x. 32398758 PMC7217864

[22] Liu Y, Luo Z-Q. 2007. The *Legionella pneumophila* effector SidJ is required for efficient recruitment of endoplasmic reticulum proteins to the bacterial phagosome. Infect Immun 75:592–603. doi:10.1128/IAI.01278-06. 17101649 PMC1828518

[23] Dalebroux ZD, Edwards RL, Swanson MS. 2009. SpoT governs *Legionella pneumophila* differentiation in host macrophages. Mol Microbiol 71:640–658. doi:10.1111/j.1365-2958.2008.06555.x. 19040633

[24] Creasey EA, Isberg RR. 2012. The protein SdhA maintains the integrity of the *Legionella*-containing vacuole. Proc Natl Acad Sci USA 109:3481–3486. doi:10.1073/pnas.1121286109. 22308473 PMC3295292

[25] Roy CR, Berger KH, Isberg RR. 1998. *Legionella pneumophila* DotA protein is required for early phagosome trafficking decisions that occur within minutes of bacterial uptake. Mol Microbiol 28:663–674. doi:10.1046/j.1365-2958.1998.00841.x. 9632267

[26] Sturgill-Koszycki S, Swanson MS. 2000. *Legionella pneumophila* replication vacuoles mature into acidic, endocytic organelles. J Exp Med 192:1261–1272. doi:10.1084/jem.192.9.1261. 11067875 PMC2193360

[27] Benz J, Meinhart A. 2014. Antibacterial effector/immunity systems: it’s just the tip of the iceberg. Curr Opin Microbiol 17:1–10. doi:10.1016/j.mib.2013.11.002. 24581686

[28] Altindis E, Dong T, Catalano C, Mekalanos J. 2015. Secretome analysis of *Vibrio cholerae* type VI secretion system reveals a new effector-immunity pair. mBio 6:e00075-15. doi:10.1128/mBio.00075-15. 25759499 PMC4453574

[29] Kirchberger PC, Unterweger D, Provenzano D, Pukatzki S, Boucher Y. 2017. Sequential displacement of type VI secretion system effector genes leads to evolution of diverse immunity gene arrays in *Vibrio cholerae*. Sci Rep 7:45133. doi:10.1038/srep45133. 28327641 PMC5361080

[30] Yang X, Long M, Shen X. 2018. Effector–immunity pairs provide the T6SS nanomachine its offensive and defensive capabilities. Molecules 23:1009. doi:10.3390/molecules23051009. 29701633 PMC6099711

[31] Feldman MF, Cornelis GR. 2003. The multitalented type III chaperones: all you can do with 15 kDa. FEMS Microbiol Lett 219:151–158. doi:10.1016/S0378-1097(03)00042-9. 12620614

[32] Belyi Y. 2020. Targeting eukaryotic mRNA translation by *Legionella pneumophila*. Front Mol Biosci 7:80. doi:10.3389/fmolb.2020.00080. 32411722 PMC7201127

[33] Ganoza MC, Kiel MC, Aoki H. 2002. Evolutionary conservation of reactions in translation. Microbiol Mol Biol Rev 66:460–485. doi:10.1128/MMBR.66.3.460-485.2002. 12209000 PMC120792

[34] Leon JAD, Qiu J, Nicolai CJ, Counihan JL, Barry KC, Xu L, Lawrence RE, Castellano BM, Zoncu R, Nomura DK, Luo Z-Q, Vance RE. 2017. Positive and negative regulation of the master metabolic regulator mTORC1 by two families of *Legionella pneumophila* effectors. Cell Rep 21:2031–2038. doi:10.1016/j.celrep.2017.10.088. 29166595 PMC5726772

[35] Belyi Y, Tartakovskaya D, Tais A, Fitzke E, Tzivelekidis T, Jank T, Rospert S, Aktories K. 2012. Elongation factor 1A is the target of growth inhibition in yeast caused by *Legionella pneumophila* glucosyltransferase Lgt1. J Biol Chem 287:26029–26037. doi:10.1074/jbc.M112.372672. 22685293 PMC3406686

[36] Belyi Y, Tabakova I, Stahl M, Aktories K. 2008. Lgt: a family of cytotoxic glucosyltransferases produced by *Legionella pneumophila*. J Bacteriol 190:3026–3035. doi:10.1128/JB.01798-07. 18281405 PMC2293231

[37] Sonesson A, Jantzen E, Bryn K, Larsson L, Eng J. 1989. Chemical composition of a lipopolysaccharide from *Legionella pneumophila*. Arch Microbiol 153:72–78. doi:10.1007/BF00277544. 2610584

[38] Frick DN, Townsend BD, Bessman MJ. 1995. A novel GDP-mannose mannosyl hydrolase shares homology with the MutT family of enzymes. J Biol Chem 270:24086–24091. doi:10.1074/jbc.270.41.24086. 7592609

[39] Guerin ME, Kaur D, Somashekar BS, Gibbs S, Gest P, Chatterjee D, Brennan PJ, Jackson M. 2009. New insights into the early steps of phosphatidylinositol mannoside biosynthesis in mycobacteria: PimB′ is an essential enzyme of Mycobacterium smegmatis. J Biol Chem 284:25687–25696. doi:10.1074/jbc.M109.030593. 19638342 PMC2757970

[40] Dover LG, Cerdeño-Tárraga AM, Pallen MJ, Parkhill J, Besra GS. 2004. Comparative cell wall core biosynthesis in the mycolated pathogens, *Mycobacterium tuberculosis* and *Corynebacterium diphtheriae*. FEMS Microbiol Rev 28:225–250. doi:10.1016/j.femsre.2003.10.001. 15109786

[41] Qaidi SE, Scott NE, Hardwidge PR. 2021. Arginine glycosylation enhances methylglyoxal detoxification. Sci Rep 11:3834. doi:10.1038/s41598-021-83437-0. 33589708 PMC7884692

[42] Qaidi SE, Scott NE, Hays MP, Geisbrecht BV, Watkins S, Hardwidge PR. 2020. An intra-bacterial activity for a T3SS effector. Sci Rep 10:1073. doi:10.1038/s41598-020-58062-y. 31974499 PMC6978387

[43] Qaidi SE, Scott NE, Hays MP, Hardwidge PR. 2022. Arginine glycosylation regulates UDP-GlcNAc biosynthesis in *Salmonella enterica*. Sci Rep 12:5293. doi:10.1038/s41598-022-09276-9. 35351940 PMC8964723

[44] Hasan MK, Qaidi SE, Hardwidge PR. 2021. The T3SS effector protease NleC is active within *Citrobacter rodentium*. Pathogens 10:589. doi:10.3390/pathogens10050589. 34065796 PMC8151275

[45] Bertram R, Schuster CF. 2014. Post-transcriptional regulation of gene expression in bacterial pathogens by toxin-antitoxin systems. Front Cell Infect Microbiol 4:6. doi:10.3389/fcimb.2014.00006. 24524029 PMC3905216

[46] Aertsen A, Michiels CW. 2004. Stress and how bacteria cope with death and survival. Crit Rev Microbiol 30:263–273. doi:10.1080/10408410490884757. 15646400

[47] Gotfredsen M, Gerdes K. 1998. The *Escherichia coli relBE* genes belong to a new toxin–antitoxin gene family. Mol Microbiol 29:1065–1076. doi:10.1046/j.1365-2958.1998.00993.x. 9767574

[48] Christensen SK, Mikkelsen M, Pedersen K, Gerdes K. 2001. RelE, a global inhibitor of translation, is activated during nutritional stress. Proc Natl Acad Sci USA 98:14328–14333. doi:10.1073/pnas.251327898. 11717402 PMC64681

[49] Machtens DA, Willerding JM, Eschenburg S, Reubold TF. 2020. Crystal structure of the metaeffector MesI (Lpg2505) from *Legionella pneumophila*. Biochem Biophys Res Commun 527:696–701. doi:10.1016/j.bbrc.2020.05.027. 32423822

[50] Hugoson E, Guliaev A, Ammunét T, Guy L. 2022. Host-adaptation in Legionellales is 1.9 Ga, coincident with eukaryogenesis. Mol Biol Evol 39:msac037. doi:10.1093/molbev/msac037. 35167692 PMC8896642

[51] Feeley JC, Gibson RJ, Gorman GW, Langford NC, Rasheed JK, Mackel DC, Baine WB. 1979. Charcoal-yeast extract agar: primary isolation medium for *Legionella pneumophila*. J Clin Microbiol 10:437–441. doi:10.1128/jcm.10.4.437-441.1979. 393713 PMC273193

[52] Saito A, Rolfe RD, Edelstein PH, Finegold SM. 1981. Comparison of liquid growth media for *Legionella pneumophila*. J Clin Microbiol 14:623–627. doi:10.1128/jcm.14.6.623-627.1981. 7037831 PMC274010

[53] Merriam JJ, Mathur R, Maxfield-Boumil R, Isberg RR. 1997. Analysis of the *Legionella pneumophila fliI* gene: intracellular growth of a defined mutant defective for flagellum biosynthesis. Infect Immun 65:2497–2501. doi:10.1128/iai.65.6.2497-2501.1997. 9169800 PMC175352

[54] Folly-Klan M, Alix E, Stalder D, Ray P, Duarte LV, Delprato A, Zeghouf M, Antonny B, Campanacci V, Roy CR, Cherfils J. 2013. A novel membrane sensor controls the localization and ArfGEF activity of bacterial RalF. PLoS Pathog 9:e1003747. doi:10.1371/journal.ppat.1003747. 24244168 PMC3828167

[55] Geisbrecht BV, Bouyain S, Pop M. 2006. An optimized system for expression and purification of secreted bacterial proteins. Protein Expr Purif 46:23–32. doi:10.1016/j.pep.2005.09.003. 16260150

[56] Bardill JP, Miller JL, Vogel JP. 2005. IcmS-dependent translocation of SdeA into macrophages by the *Legionella pneumophila* type IV secretion system. Mol Microbiol 56:90–103. doi:10.1111/j.1365-2958.2005.04539.x. 15773981

[57] Blakeley BD, Chapman AM, McNaughton BR. 2012. Split-superpositive GFP reassembly is a fast, efficient, and robust method for detecting protein-protein interactions *in vivo*. Mol Biosyst 8:2036–2040. doi:10.1039/c2mb25130b. 22692102

[58] Lara-Tejero M, Sutterwala FS, Ogura Y, Grant EP, Bertin J, Coyle AJ, Flavell RA, Galán JE. 2006. Role of the caspase-1 inflammasome in *Salmonella typhimurium* pathogenesis. J Exp Med 203:1407–1412. doi:10.1084/jem.20060206. 16717117 PMC2118315

[59] Case CL, Roy CR. 2013. Analyzing caspase-1 activation during *Legionella pneumophila* infection in macrophages. Methods Mol Biol 954:479–491. doi:10.1007/978-1-62703-161-5_29. 23150415

[60] Arasaki K, Roy CR. 2010. *Legionella pneumophila* promotes functional interactions between plasma membrane syntaxins and Sec22b. Traffic 11:587–600. doi:10.1111/j.1600-0854.2010.01050.x. 20163564 PMC3164831

