# Supplementary figures and images for: Intrabacterial Regulation of a Cytotoxic Effector by Its Cognate Metaeffector Promotes Legionella pneumophila Virulence
Source: mSphere. 2023 Jan 4;8(1):e00552-22. doi: 10.1128/msphere.00552-22 (PMC9942577; doi:10.1128/msphere.00552-22)

*In vitro*

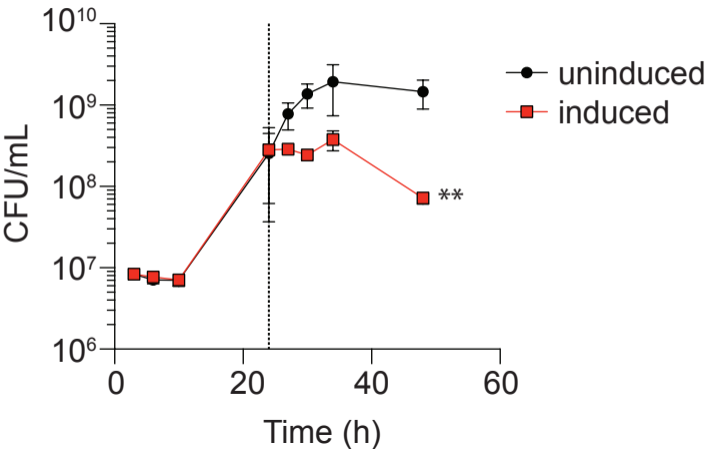

Supplement: FIG S1 [file msphere.00552-22-s0001.pdf]

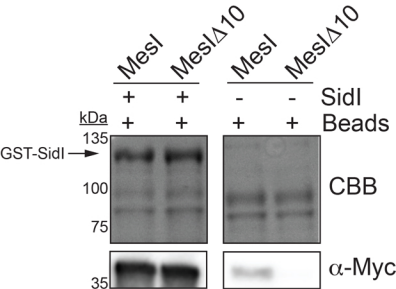

Supplement: FIG S2 [file msphere.00552-22-s0002.pdf]

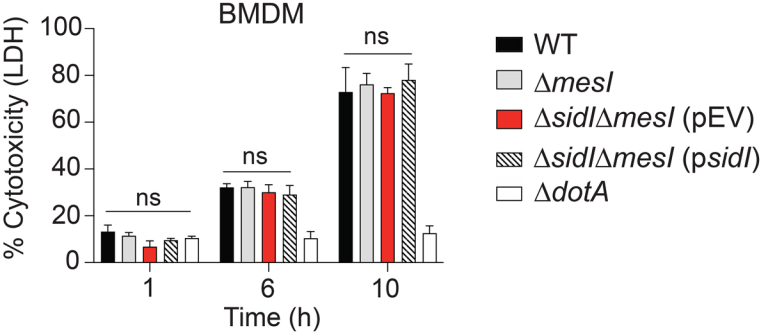

Supplement: FIG S3 [file msphere.00552-22-s0003.pdf]
